# Long Noncoding RNA SCIRT Promotes HUVEC Angiogenesis via Stabilizing VEGFA mRNA Induced by Hypoxia

**DOI:** 10.1155/2022/9102978

**Published:** 2022-06-03

**Authors:** Lianze Gao, Jihong Yang, Yi Li, Keyu Liu, Huilin Sun, Jing Tang, Zhengyuan Xia, Liangqing Zhang, Zhe Hu

**Affiliations:** ^1^Department of Anesthesiology, Affiliated Hospital of Guangdong Medical University, Zhanjiang 524001, China; ^2^Department of Medicine, Columbia Center for Human Development, Columbia University Irving Medical Center, New York, NY 10032, USA

## Abstract

Ischemia-reperfusion injury (IRI) is closely associated the abnormal expression of long noncoding RNAs (lncRNAs), especially for their regulatory roles in IRI-related angiogenesis. This study applied a hypoxia-reoxygenation (HR) cell model to simulate the IRI condition, as well as RNA sequencing and RNA pull-down experiments to reveal roles of the lncRNA and Stem Cell Inhibitory RNA Transcript (SCIRT), in endothelial angiogenesis. We found that SCIRT was increased under the HR condition and exhibited a high expression correlation with angiogenesis marker VEGFA. RNA-seq data analysis further revealed that VEGFA-related angiogenesis was regulated by SCIRT in HUVECs. Gain and loss of function experiments proved that SCIRT posttranscriptionally regulated VEGFA via affecting its mRNA stability. Furthermore, HuR (ELAVL1), an RNA binding protein (RBP), was identified as a SCIRT-binding partner, which bound and stabilized VEGFA. Moreover, SCIRT promoted HuR expression posttranslationally by inhibiting its ubiquitination under the HR condition. These findings reveal that lncRNA SCIRT can mediate endothelial angiogenesis by stabilizing the VEGFA mRNA via modulating RBP HuR stability under the HR condition.

## 1. Introduction

The reperfusion of the bloodstream to an organ in ischemia is essential for its functional recovery; however, the rapid influx of blood oxygen will cause additional lesions, known as ischemia-reperfusion injury (IRI) [1, 2]. Mammalian cells dynamically change their gene expression patterns in response to the hypoxic challenge by triggering adaptive processes, such as halting cell division, undergoing apoptosis, accelerating proliferation. These changes always occur via transcriptional control or posttranscriptional regulation [3]. The main posttranscriptional processes, including mRNA turnover and translational control, are modulated efficiently by microRNAs, RNA-binding proteins (RBPs), and long noncoding RNAs (lncRNAs) [4–6].

lncRNAs are transcripts with length more than 200 nt. These transcripts own a limited coding potential but can function at multiple levels, including mRNA splicing, translation, and degradation [7, 8]. With varying levels of evidence, lncRNAs have been implicated in cancers, developmental defects, neurodegenerative, and cardiovascular diseases [9, 10]. Furthermore, lncRNAs play crucial roles in myocardial infarction (MI) and myocardial IRI [4, 11]. For example, inhibition of the lncRNA H19 abated oxidative stress and inflammation *in vitro*. Moreover, ablation of H19 could improve cardiac function and decrease infarct size in myocardial IRI mice [12]. In addition to cardiomyocytes, endothelium cells (ECs) in the vascular system are sensitive to IRI. Hypoxia condition changes the permeability properties of ECs, resulting in endothelial dysfunction [13], as characterized by reduction of nitric oxide secretion [14], essential vasodilators, and ROS production [15, 16], thus dysregulating vascular homeostasis. Increasing evidence has been shown for the therapeutic potential of lncRNAs in treating endothelial dysfunction in heart diseases [17–19], especially in therapeutic angiogenesis after myocardial infarction [20–22]. These findings strongly suggest the endothelial model as a highly efficient model for exploring therapeutic lncRNAs for treating IRI-related endothelial dysfunction.

In this study, we mimicked the *in vivo* myocardial IRI using a hypoxia-reoxygenation- (HR-) induced human umbilical vein endothelial cell (HUVEC) model. A global transcriptome analysis on this model identified Stem Cell Inhibitory RNA Transcript (SCIRT) as the top lncRNA that was enriched in HR condition, as well as showing high coexpression trend with an angiogenesis marker gene vascular endothelial growth factor A (VEGFA). We further utilized RNA-sequencing and RNA-pull down experiments to investigate potential mechanisms of SCIRT in oxidative stress-induced vascular endothelial cell injury. We found that the upregulated SCIRT expression by HR increased VEGFA protein level by stabilizing its mRNA. In particular, SCIRT was found to interact with HuR and posttranslationally upregulated the HuR protein level by decreasing its ubiquitination under HR condition. The increased HuR further enhanced the stability of VEGFA mRNA, thus promoting therapeutic angiogenesis. Thus, the present study provides a potential target for endothelial cells in the context of ischemia-reperfusion injury.

## 2. Materials and Methods

### 2.1. HUVEC Culture

HUVECs were obtained from the cell bank of type culture collection of the Chinese academy of sciences, Shanghai, China. Briefly, HUVECs were cultured at 37°C, in a 95% O_2_ and 5% CO_2_ humidified atmosphere in DMEM supplemented with 10% fetal bovine serum, 100 *μ*g/ml streptomycin, and100 IU/ml penicillin. The cultured HUVECs were incubated under hypoxic condition (5% CO_2_ and 95% N_2_) for 12 h, followed with reoxygenation (HR) for 4 h to establish in vitro HR model.

### 2.2. Plasmid Construction and Cell Transfection

The whole length of SCIRT or HuR was amplified by PCR and subcloned into pcDNA3.1 vector (GenePharma, Shanghai, China). Specific siRNA (GenePharma, Shanghai, China) or pcDNA3.1-SCIRT/pcDNA3.1-HuR were transfected into cells with Lipofectamine RNAiMAX (Thermo Fisher Scientific) and Lipofectamine 3000 (Thermo Fisher Scientific), respectively. After 36 h of transfection, the cells were incubated under HR condition for another continuous 16 h, which were further collected for RNA or protein extraction. The siRNA sequences are listed in Table I.

### 2.3. Evaluation of Cell Angiogenic Ability

For wound healing assays, HUVECs transfected with corresponding siRNAs were planted in 6-well plates and cultured to form single confluent cell layers. Then, a 200 *μ*l pipette tip was used to scratch the cell layers, and a wound was generated. Pictures were taken at the timepoint of 0 h and 24 h.

Transfected cells (transfected for 36 h) were treated under hypoxic condition for 12 h, followed with reoxygenation (HR) for 4 h, then used for transwell assays. The experiments were carried out using Corning transwell chambers; 1 × 10^5^ treated cells suspended in 200 *μ*l medium (without serum) were placed into the upper sides of transwell chambers. A lower chamber was filled with 600 *μ*l medium (with 15% FBS). After incubation for 24 h at 37°C with 5% CO_2_, the invaded cells were stained with crystal violet and photographed.

Tube formation assay was performed to detect the angiogenic ability of HUVECs. In brief, cells were planted at a density of 2 × 10^4^ on 96-well plates coated with 150 *μ*l Matrigel. Being cultured for 4-6 h at 37°C, the average number of tubes was counted in 5 random microscopic fields with a computer-assisted microscope.

### 2.4. Western Blotting

The expression levels of VEGFA and HuR were determined by western blot analysis. Cells were harvested after HR treatment; the whole cell lysates were prepared in RIPA buffer (50 mM Tris HCl, pH 7.4, 150 mM NaCl, 1% sodium deoxycholate, 0.1% SDS, and 1% Triton X-100 plus proteinase inhibitors; SANTA CRUZ). Protein concentration was determined by BCA assay (Beyotime, Guangzhou, China), and 30 *μ*g protein samples were separated by 12% SDS-PAGE. Proteins were transferred to Millipore polyvinylidene difluoride (PVDF) membranes. After being blocked in 5% BSA for 1 h at room temperature, the membranes were then incubated overnight at 4°C with different primary monoclonal antibodies; GAPDH and Tubulin antibody (Bioworld) were used as a loading control. The membranes were then incubated with secondary antibody for 1 h at room temperature and proceed to be exposed with enhanced chemiluminescent reagents. Next, the proteins were detected using a Tanon 5200 scanner.

### 2.5. RNA Purification and Real-Time Quantitative RT-PCR (qRT-PCR)

Total RNA was extracted from HUVECs using TRIzol reagent (Ambion), treated with Recombinant DNase I (Takara). The isolated total RNA was reverse transcribed into cDNA using a TakaRa PrimeScript™ RT reagent Kit. Then, the qRT-PCR analysis was carried out using a SYBR Green qPCR Master Mix (RR820A Takara) on a ROCHE LightCycler 480II Real-time PCR apparatus. GAPDH was used as the endogenous control. RNA levels were quantified using the methods of 2-*ΔΔ*Ct. The primer sequences are listed in Table II.

### 2.6. Fluorescent In Situ Hybridization (FISH)

FISH was conducted using Fluorescent In Situ Hybridization Kit (RIBOBIO, Guangzhou, China) according to the manufacturer's recommendations. Probes labeled with Quasar570 were used to target SCIRT transcripts. Briefly, 1 × 10^4^ HUVECs were cultured on 24-well plates (Corning) for 24 h, then washed with PBS, and fixed with 4% paraformaldehyde for 15 min. After PBS washing and 70% ethanol permeabilization at 4°C for 1 h, probe hybridization was conducted at 37°C overnight in the incubator. Finally, cells were counterstained with DAPI (blue), and images were acquired with an OLYMPUS DP71 fluorescence microscope.

### 2.7. RNA Pull-Down

RNA pull-down was performed as described [23]. Briefly, biotinylated RNAs were transcribed in vitro by T7 polymerase according to the manufacturer's protocol (Thermo Fisher Scientific). 50 *μ*l of magnetic beads was used to incubate with 100 pmol RNA for 30 minutes at room temperature with agitation. Beads were then mixed with cell lysate and incubated in protein-RNA binding buffer overnight at 4°C. To harvest the protein complex, 50 *μ*l 5×SDS loading buffer was added and boiled for 10 min at 95°C. Retrieved proteins were analyzed by western blot with anti-HuR antibody (MA1-167, Thermo Fisher Scientific).

### 2.8. RNA Immunoprecipitation (RIP)

RNA IP (RIP) for HuR protein was performed using RNA-Binding Protein Immunoprecipitation Kit (Cat.#17-700, Millipore) according to the manufacturer's instructions. Cell lysates were prepared with RIP lysis buffer and then incubated with anti-HuR antibodyor normal mouse IgG (ab65986, Abcam) at 4°C for 6 h with rotation. RNA-protein complexes were retrieved by magnetic beads (Magnetic Beads Protein A/G, CS203178), washed 6 times in the cold RIP wash buffer. After proteinase K treatment, beads were separated using a magnetic separator from the sample. The supernatant was directly resuspended in TRIzol reagent and subjected to RNA extraction. Then, qRT-PCR analysis was performed, and the RNA levels in RIP samples were normalized to input samples.

### 2.9. Ubiquitination Assay

Ub IP for ubiquitination assay was carried by Co-Immunoprecipitation (Co-IP) Kit (Cat. #26149, Pierce). Cells treated with MG132 and transfected with siRNA were prepared in RIPA buffer. Then, the cell lysates were incubated with 10 *μ*g anti-Ubiquitin antibody (1862775, Thermo Fisher Scientific) or normal mouse IgG and 50 *μ*l Protein A + G beads at 4°C for 2 h with rotation. After the wash with cold PBS for 3 times, the ubiquitinated proteins were retrieved by eluting in 50 *μ*l 2×SDS loading buffer and subjected to western blot.

### 2.10. RNA Stability Assays

HUVECs were treated with actinomycin D at the concentration of 5 *μ*g/ml. The cells were harvested at indicated time points, and cell lysates were prepared with RIP lysis buffer and subjected to western blot.

### 2.11. Microarray and RNA-seq Data Analysis

For microarray data, the raw data were first log_2_-normalized and then used to evaluate the statistical significance between different groups using the limma [24] in R software.

For RNA-seq data, we first aligned our RNA-seq read data to hg19 genome using Bowtie2 (v2.3.4.3) [25], and aligned bam files were sorted by name using the parameter -*n*. We used the HTSeq software (v0.11.2) [26] and hg19 annotation file from GENCODE [27] to count reads for each gene using parameters -*r*name -*f*bam –stranded = reverse, and BioMart [28] to retrieve corresponding gene names. Finally, read counts were normalized with the trimmed mean of *M*-value (TMM) method [29] for differential expression analysis using edgeR (v3.26.8) [30].

### 2.12. Statistical Analysis

In addition to statistical analysis on microarray and RNA-seq data described above, we used GraphPad Prism 7.0 for statistical analysis on other data included in the study. Quantitative data were expressed as the mean ± standard deviation (SD). The variables between groups were compared by Student's *t*-test or one-way analysis of variance (ANOVA) with post hoc Tukey's tests. *p* value < 0.05 was considered statistically significant.

## 3. Result

### 3.1. HR Promotes HUVEC Angiogenesis with the Marker VEGFA Upregulated

**Figure 1:**
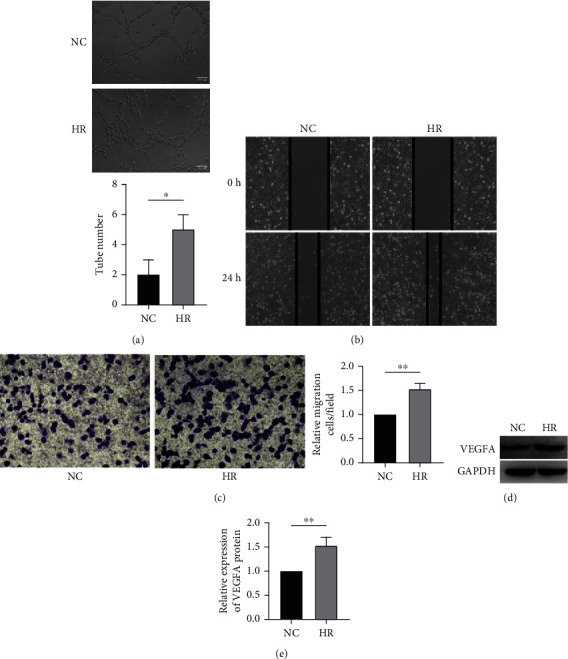
HR promotes HUVEC angiogenesis with the marker VEGFA upregulated. (a) Representative images of the tube formation assay (scale bar, 100 *μ*m). (b) Representative images of the wound healing assay. (c) Representative images of transwell migration assay. (d) Protein levels of VEGFA in NC and HR conditions using western blot analysis. (e) Quantitative analysis of (d). The data showed that HR treatment induced the tube formation and promoted the migration of HUVECs. The ratio of invaded cells in the HR group was significantly higher than that in the NC group. VEGFA protein level is increased in the HR group compared with the NC group in HUVECs. (e) Quantitative analysis of (d). HR: hypoxia-reoxygenation; NC: negative control; HUVEC: human umbilical vein endothelial cell; ^∗^*p* < 0.05,  ^∗∗^*p* < 0.01,  ^∗∗∗^*p* < 0.001, and^∗∗∗∗^*p* < 0.0001.

HR-treated cells were obtained as previously described [31]. Briefly, HUVECs were first cultured under hypoxia condition for 12 h and followed by 4 h reoxygenation. Tube formation, wound healing, and transwell migration assay (Figures 1(a)–1(c)) were used to evaluate the angiogenic and migration capacity. Both capacities were remarkably enhanced with the HR treatment. We also found the expression of VEGFA, a well-known angiogenesis marker and critical regulator of blood vessel formation and maintenance [32], was consistently upregulated in HR-treated cells (Figures 1(d) and 1(e)).

### 3.2. LNC Is Identified as a Positive Regulator of Angiogenic Effect under HR Condition

**Figure 2:**
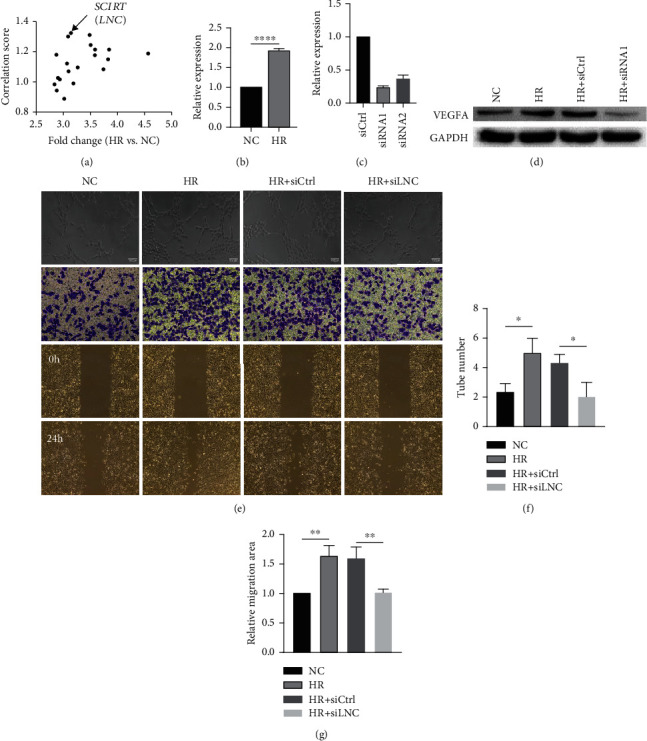
SCIRT is identified as a positive regulator of angiogenic effect under HR condition. (a) SCIRT exhibited the highest correlation score within top 20 upregulated lncRNAs in the HR vs. NC group. (b) qPCR validation of increased SCIRT expression in the HR vs. NC group. (c) Relative expression levels of SCIRT in HUVECs after transfection of SCIRT siRNAs (siRNA1 and siRNA2) and control siRNAs (siCtrl). (d) Inhibition of SCIRT downregulates the protein levels of VEGFA. HUVECs were transfected with SCIRT siRNA, the expression of VEGFA was analyzed by western blot. (e) Tube formation, migration and invasion assay demonstrated that angiogenesis ability was decreased by the knockdown of SCIRT in HUVECs (scale bar, 100 *μ*m). Quantitative analysis of (f) tube formation assay and (g) wound healing assay, respectively. ^∗^*p* < 0.05,  ^∗∗^*p* < 0.01,  ^∗∗∗^*p* < 0.001, and^∗∗∗∗^*p* < 0.0001.

Gene microarray was applied to compare HR-treated with untreated control HUVECs. Among the top 20 significantly upregulated lncRNAs in the HR-treated vs. control group, SCIRT, a reported tumor repressor [33], exhibited the highest angiogenesis correlation score (Figure 2(a)). The increase of SCIRT induced by HR treatment was further confirmed by qRT-PCR (Figure 2(b)). Since the high angiogenesis score of a gene depends on its positive correlation with an angiogenesis marker VEGFA [34, 35] and negative correlation with an angiogenesis suppressor THBS1 [36, 37], we wonder if SCIRT participates in HUVEC angiogenesis through regulating VEGFA or THBS1. siRNAs were designed to knock down SCIRT, and efficiency was confirmed by qRT-PCR (Figure 2(c)). RNA sequencing was then used to obtain the transcriptome profiles of HUVECs under normal (NC), HR-treated (HR), and SCIRT knockdown (KD) conditions. A total of 2789 genes, called HR enriched genes, were significantly upregulated in HR vs. NC conditions, of which 649 genes were downregulated by SCIRT knockdown (FigS1). Interestingly, gene ontology (GO) analysis indicated these 649 genes enriched with VEGF-related angiogenesis (FigS1). As expect, SCIRT depletion led to a decrease of VEGFA in protein level (Figure 2(d)). Moreover, SCIRT knockdown also reduced the angiogenic and migration capacity of HUVECs (Figures 2(e)–2(g)).

### 3.3. LNC OE Further Increases the Angiogenesis Ability via Stabilizing VEGFA mRNA under HR Condition

**Figure 3:**
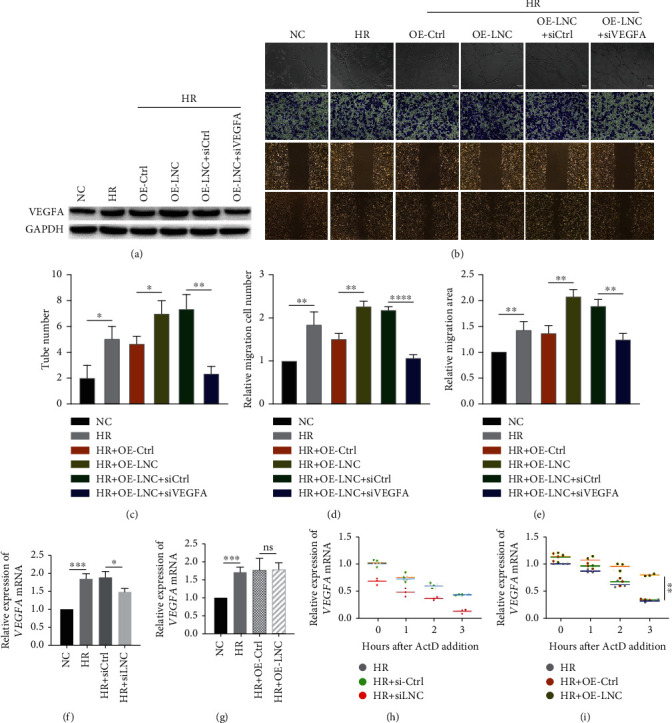
SCIRT increases the angiogenesis via stabilizing VEGFA mRNA under HR condition. (a) SCIRT upregulated VEGFA with HR treated in HUVECs, which was inhibited when cells transfected with siRNA targeting VEGFA. Protein levels were determined by western blot. (b) Tube formation assay, transwell migration assay, and wound healing assay were employed to evaluate the angiogenesis ability of HUVECs, which was increased with ectopic expression of SCIRT, while being repressed with the inhibition of VEGFA (scale bar, 100 *μ*m). Quantitative analyses of tube formation assay, wound healing assay, and transwell migration assay are shown in (c–e), respectively. (f) Inhibition of SCIRT downregulates VEGFA mRNA expression. (g) No alterations of VEGFA mRNA level were observed as ectopic expression of SCIRT. (h) Cells were treated with ActD and harvested at various time points for mRNA half-life assay; RNA levels were detected by qPCR. VEGFA mRNA stability was not affected by knockdown of SCIRT. (i) Cells were treated with ActD and harvested at various time points for mRNA half-lives assay; RNA levels were detected by qPCR. VEGFA mRNA stability was shown to be elevated by overexpression of SCIRT. ^ns^*p* > 0.05,  ^∗^*p* < 0.05,  ^∗∗^*p* < 0.01,  ^∗∗∗^*p* < 0.001, and^∗∗∗∗^*p* < 0.0001.

Next, we studied whether SCIRT overexpression will affect the VEGFA expression in HUVECs. The protein level of VEGFA was elevated by SCIRT overexpression and repressed by siRNA target VEGFA in HUVECs under the HR condition (Figure 3(a), FigS2). The angiogenesis ability was enhanced by SCIRT overexpression and inhibited by VEGFA repression (Figures 3(b)–3(e)). These data suggested that SCIRT might positively regulate VEGFA under the HR condition. We also checked the mRNA level of VEGFA; SCIRT knockdown decreased VEGFA (Figure 3(f)). However, SCIRT overexpression failed to alter the VEGFA mRNA level (Figure 3(g)). VEGFA was reported to be induced by hypoxia due to an increase in its mRNA stability [38]. No change of VEGFA mRNA level by SCIRT overexpression indicated that VEGFA mRNA had already been mainly stabilized under HR condition. Thus, we turned to check the mRNA stability of VEGFA by blocking the RNA transcription using actinomycin D. Under the HR condition, the knockdown of SCIRT reduced the expression of VEGFA, but there was no difference at each time point (Figure 3(h)); however, SCIRT overexpression slowed down the degradation rate of VEGFA, suggesting that SCIRT might regulate the VEGFA mRNA stability (Figure 3(i)).

### 3.4. LNC Interacts with HuR to Stabilize the VEGFA through Ubiquitin-Proteasome Pathway under HR Condition

**Figure 4:**
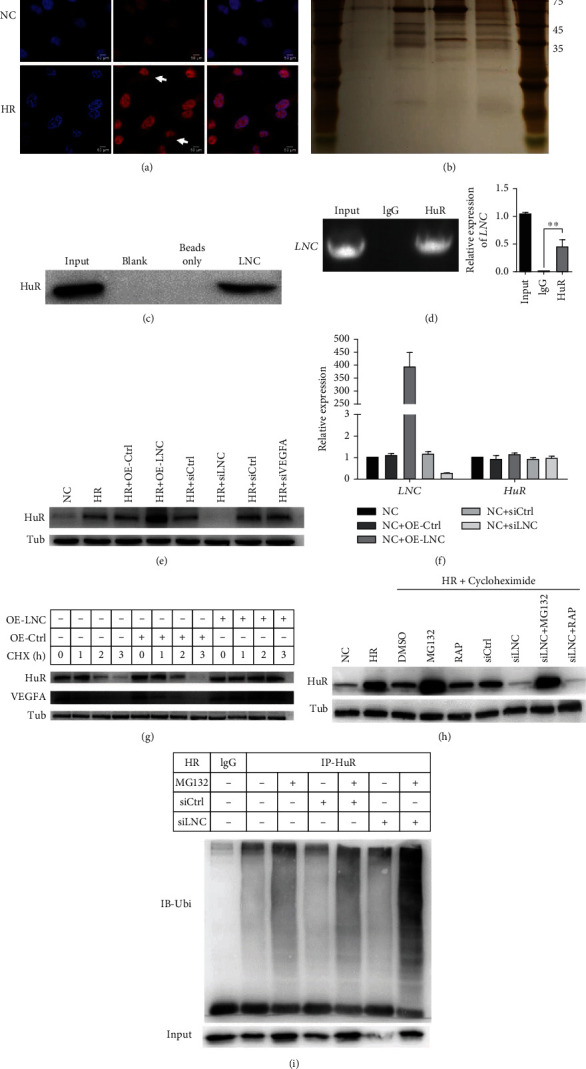
SCIRT interacts with HuR to stabilize the VEGFA mRNA through ubiquitin-proteasome pathway under HR condition. (a) RNA FISH revealed that SCIRT is predominantly localized in the nuclear of HUVECs, while being exported to the cytoplasm with HR treatment (scale bar, 50 *μ*m). (b) Identification of proteins that associate with SCIRT. Biotinylated SCIRT or biotinylated antisense SCIRT was incubated with HUVEC extracts; RNA pull-down combined was performed to identify the proteins that associate with SCIRT. (c) HuR was identified specifically bind to SCIRT by western blot. (d) RIP was performed to confirm the interaction of HuR and SCIRT. (e) The protein level of HuR determined by western blot was positively regulated by SCIRT, however, which was not affected by variation of VEGFA. (f) Overexpression and knocking down of SCIRT have no effect on RNA levels of HuR; HuR was regulated by SCIRT post-translationally. (g) HUVECs, transfected with the siRNA of SCIRT, were treated with CHX (125 *μ*g/ml), an inhibitor of protein synthesis. The protein levels of HuR and VEGFA were detected by western blot after 1, 2, and 3 h. (h) Western blot analysis of HuR in the SCIRT knockdown cells treated with DMSO, MG132, or RAP under HR conditions. (i) HUVECs transfected with SCIRT siRNA were treated with MG132 (5 *μ*M) for 24 h. Cell lysates were immunoprecipitated with either control IgG or an antibody against HuR and analyzed by immunoblotting with a ubiquitin- (Ub-) specific antibody. Bottom, input from cell lysates.

To further explore the regulation mode of SCIRT, RNA fluorescence in situ hybridization (FISH) was used to identify its subcellular localization, which is important for exploring the function of lncRNAs. SCIRT was typically detected mainly localized in nuclei of HUVECs, while being exported to the cytoplasm after the HR treatment (Figure 4(a)). Some cytoplasm-localized lncRNAs can regulate genomic stability by sequestering proteins [39]. To determine the mechanism by which SCIRT modulates the activity of VEGFA, we sought to identify the RNA binding proteins of SCIRT through the RNA pull-down assay (Figure 4(b)). Our result suggested that SCIRT might regulate the VEGFA mRNA stability and that HuR was reported to bind VEGFA and enhanced its mRNA stability by hypoxia [38]; so we chose the HuR as a candidate for western blot following RNA pull-down assay. HuR was detected to bind SCIRT in western blot results (Figure 4(c)), and the selective binding of HuR with SCIRT was further supported by RNA immunoprecipitation (RIP) results (Figure 4(d)).

Under the HR condition, the HuR protein level was remarkably increased by SCIRT overexpression while decreased by SCIRT inhibition. However, VEGFA knockdown did not affect HuR expression, indicating that SCIRT might be upstream of HuR under the HR condition (Figure 4(e)). We further checked the HuR RNA level, and neither SCIRT ectopic overexpression nor repression changed the HuR RNA level (Figure 4(f)). To understand how SCIRT modulates HuR protein expression, we first treated HUVECs with a protein synthesis inhibitor cycloheximide (CHX). We found that SCIRT ectopic overexpression significantly inhibited HuR protein degradation, suggesting that SCIRT posttranslationally regulated HuR protein expression (Figure 4(g)). Protein ubiquitination modification is one of the critical posttranslational ways of modulating protein levels [40]. To figure out how SCIRT regulates HuR protein levels through ubiquitination, MG132, a specific proteasome inhibitor, was employed to treat HUVECs. Downregulation of HuR protein was abolished upon SCIRT knockdown (Figure 4(h)), indicating that HuR was a proteasome substrate. Thus, SCIRT posttranslationally regulated HuR protein expression through modulating HuR degradation. Finally, we investigated the mechanism of how SCIRT regulates HuR protein degradation. HuR ubiquitination level was found to increase upon knockdown of endogenous SCIRT (Figure 4(i)). These data demonstrate that SCIRT posttranslationally activates HuR protein expression by inhibiting its ubiquitination and degradation. Under the HR condition, SCIRT facilitated the accumulation of HuR proteins, then enhanced the VEGFA mRNA stability.

### 3.5. HuR Acts as a Key Downstream to Induce the Angiogenesis Effect under HR Condition

**Figure 5:**
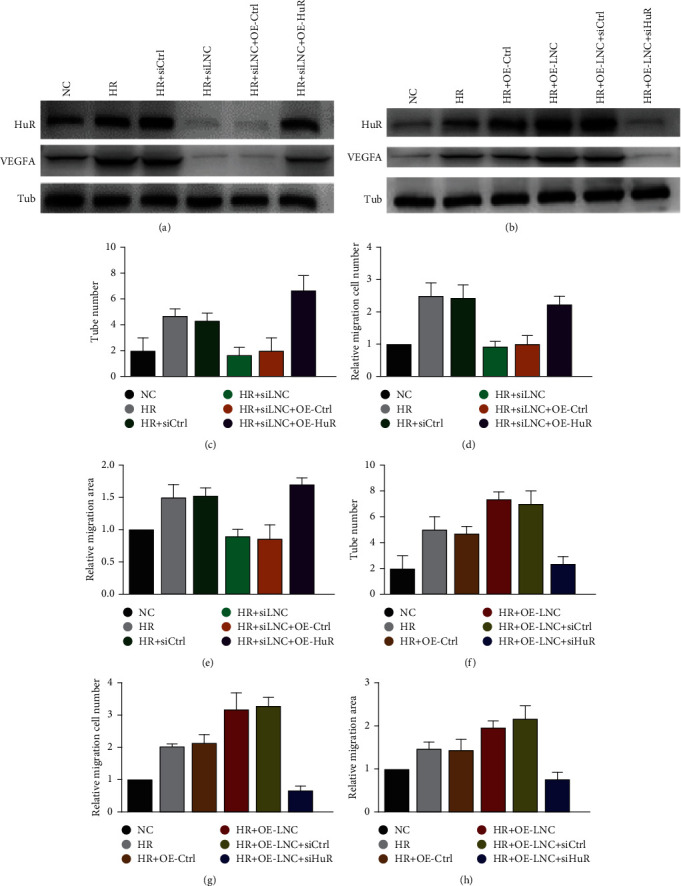
HuR acts as a key downstream factor to induce the angiogenesis effect under HR condition. (a) Inhibition of SCIRT downregulated protein levels of HuR and VEGFA, which was reversed by ectopic expression of HuR. (b) Overexpression of SCIRT upregulated protein levels of HuR and VEGFA, which can be counteracted with HuR knocked down. Protein levels were examined by western blot. Under HR condition, the cell ability of tube formation (c), migration (d), and invasion (e) ability was decreased in HUVECs transfected with SCIRT siRNA, which were rescued by ectopic expression of HuR. On the contrary, the SCIRT overexpression-induced cell angiogenesis ability, evaluated by tube formation assay (f), transwell migration assay (g), and wound-healing assay (h), was hampered by HuR inhibition.

To elucidate the mechanism of how SCIRT regulates HuR protein degradation involved in the VEGFA mRNA stability, we knocked down or overexpressed SCIRT in HR-treated HUVECs. VEGFA and HuR decreased with SCIRT knockdown but were rescued by the ectopic expression of HuR (Figure 5(a)). Furthermore, VEGFA and HuR were upregulated by SCIRT overexpression while being decreased by HuR repression (Figure 5(b)). Consistently, the migration capacity of HUVECs was enhanced by SCIRT but repressed by HuR withdrawal (Figures 5(c)–5(h), FigS3). Taken together, HuR acts as a downstream element of SCIRT to induce angiogenesis effect through stabilizing VEGFA under the HR condition.

## 4. Discussion

The endothelium, covering the innermost apical surface of all blood and lymphatic vessels, contributes to vascular homeostasis [41]. Stress-related dysfunction of EC results in diseases in the heart, kidney, and liver [42]. Ischemia-reperfusion (IR) induces deteriorative effects on either large vessels or the microcirculation of the heart. ECs were indicated in several studies to be more sensitive to IR than cardiomyocytes, which are central mediators during the progression of cardiac IR-injury (IRI) [2, 43–45]. Thus, the endothelium is an appropriate therapeutic target for protecting the myocardium from IRI via conditioning strategies or cardioprotective drugs.

In the present study, we applied an HR cell model to simulate IRI. We performed a gene chip analysis on HUVECs to explore underlying targets related to HR-associated cell injury. Further combined with western blot analysis, we found both RNA and protein levels of VEGFA increased after HR. Hypoxia is an important stimulator of VEGF expression. VEGFA can increase microvascular permeability and endothelial cell migration, thus enhancing angiogenesis in the ischemic organ and reducing IRI, becoming a research hotspot and potential target of ischemic stroke [46, 47]. Our gene chip results revealed that lncRNA SCIRT was highly correlated with VEGFA and showed a significant increase after HR. Furthermore, SCIRT KD leads to defect in VEGF-related angiogenesis as indicated by our RNA-seq data. Given these, we hypothesized that SCIRT might regulate VEGFA in angiogenesis during the process of HR.

lncRNAs, besides their roles as regulators of transcription, have been reported to regulate gene expression posttranscriptionally in a variety of ways: splicing of pre-mRNAs [8], protection or acceleration mRNA decay [48], repression or activation of mRNA translation [49], or functional association with microRNAs, which “sponges” microRNAs, promoting target mRNA translation by competing with a microRNAs [50]. In the present study, we found that SCIRT knockdown decreased VEGFA in both RNA and protein levels; while SCIRT was overexpressed, VEGFA upregulated at the protein level, but no change at the RNA level, suggesting that SCIRT posttranscriptionally regulated VEGFA. The function of lncRNAs partially depends on their subcellular localization. lncRNAs exert most of the posttranscriptional regulation in the cytoplasm. To elucidate the exact mechanism of SCIRT in HR cells, we applied FISH to detect its cellular localization. SCIRT was found exported to cytoplasm under the HR condition.

lncRNAs can interact and regulate the expression of RNA binding proteins (RBPs). The latter can modulate mRNA turnover and translation. For example, HHIP-AS1 interacted with and positively regulated the stability of HHIP mRNA in a HuR-dependent manner [51]. DANCR bound the CTNNB1 3′UTR region and blocked the repressing effect of microRNAs on CTNNB1 [52]. In our study, RBP HuR was detected binding to SCIRT by RNA pull-down assay. HuR, belonging to the embryonic lethal abnormal vision (ELAV)/Hu protein family [53], regulates cellular proliferation [54]. HuR, predominantly nuclear-localized, can translocate to cytoplasm induced by oxidative stress agents (e.g., H_2_O_2_) or by irradiation with short-wave-length ultraviolet light (UVC), which was similar to our result that SCIRT was exported to the cytoplasm after HR. In general, the functional activity of HuR depends on its dynamic subcellular localization [55, 56]. Cytoplasmic localization benefits HuR to stabilize and increase the translation of target mRNAs involved in the pathogenesis of numerous cancers and various diseases [57, 58]. Small molecule inhibitors of HuR translocation has been underutilized in the clinical setting [59–61]. The association of HuR with cofactors such as ANP32B, ANP32A, and XPO1 has been shown to be key mediators of HuR export [62–64]. lncRNAs were reported to regulate several biological processes including nuclear-cytoplasmic trafficking [65, 66]. We confirmed the interaction between SCIRT and HuR, and HR induces SCIRT nuclear export to the cytoplasm, where it regulates mRNA stability, suggesting that SCIRT is likely to cooperate with HuR to be exported to cytoplasm.

The RBP HuR can be posttranslationally modified, such as phosphorylation, ubiquitination, or methylation. Ubiquitination is an important posttranslational modification involved in a variety of cellular processes. OCC-1 was reported to regulate the level of HuR protein by promoting its ubiquitination and degradation in colorectal cancer [67]. ASB16-AS1 inhibits HuR expression post-translationally by promoting its ubiquitination in adrenocortical carcinoma [68]. In our study, SCIRT was found to bind and repress ubiquitination of HuR, thus stabilizing VEGFA mRNA. HuR can recognize and bind to specific mRNAs bearing AU-rich RNA elements (AREs) to enhance their stability, translation, or both [69, 70]. In stress-treated cells, HuR was shown to target mRNA-encoding proteins important for cell growth and proliferation, including those that encode stress-response and proliferation proteins such as p21, c-FOS, and cyclins [71].VEGFA, a potent angiogenic factor, increases significantly after ischemia, but VEGF transcripts under the normal oxygen level are short-lived and highly labile [72]. The 3′-UTR of VEGFA possesses several AU-rich elements (ARE), which is the critical region contributing to the stability of VEGFA mRNAs under hypoxia. It was reported that HuR bound VEGFA with high affinity and enhanced VEGF mRNA stability by hypoxia [38].

We further curated the HuR targets identified in HUVECs from the Nature Medicine paper [73], consisting of 2578 targets (Table III). We further analyzed these targets and found 2183 genes were measured in our RNA-seq. There were 108 (Table IV) and 169 (Table V) out of above target genes which were significantly upregulated or downregulated by lncRNA SCIRT knockdown with absolute(log_2_FC) > 1 and *p* value < 0.05, respectively. The above 169 downregulated HuR target genes by lncRNA SCIRT knockdown enriched five angiogenesis-related terms, namely, “regulation of angiogenesis (GO: 0045765, *p*-value = 0.0017),” “angiogenesis involved in wound healing (GO: 0060055, *p* value = 0.0037),” “positive regulation of cell migration involved in sprouting angiogenesis (GO: 0090050, *p* value = 0.0099),” “regulation of cell migration involved in sprouting angiogenesis (GO: 0090049, *p* value = 0.030),” and “negative regulation of angiogenesis (GO: 0016525, *p* value = 0.0038).” On the other hand, no angiogenesis-related terms were enriched by the 108 upregulated target genes. In addition to the above 277 significantly affected target genes, there are still 1906 target genes that are not significantly changed by the SCIRT knockdown. Though we showed potential connection between the regulation of SCIRT on HuR target expression and angiogenesis, it is challenging to have a comprehensive understanding of the regulatory roles of SCIRT on HuR target expression, as well as stability.

In summary, we found that SCIRT was coupregulated with VEGFA in HR-treated cells, which promoted the angiogenic ability of HUVECs. In detail, SCIRT interacted with HuR and inhibited its ubiquitination and degradation, thus positively regulating VEGFA mRNA's stability. This study explored the mechanism of SCIRT in VEGFA regulation under the hypoxia-reoxygenation condition and might provide a new therapeutic target for IRI.

## Data Availability

All high-throughput microarray and RNA-seq data generated in this study are available at the Gene Expression Omnibus under accession code GSE193047 and GSE193048. To review the microarray data, please visit the link: https://www.ncbi.nlm.nih.gov/geo/query/acc.cgi?acc=GSE193047, and enter the token etifiuwitxixbgt into the box. To review the RNA-seq data, please visit the link: https://www.ncbi.nlm.nih.gov/geo/query/acc.cgi?acc=GSE193048, and enter token qbaraqginnohxcn into the box. All mRNA and lncRNA expressions analyzed from microarray data are organized into Tables VI and VII, respectively. For RNA-seq data, the normalized expression table for all genes is in Table VIII.

## Supplementary Materials

Supplementary 1 Materials FigS1: transcriptional changes induced by SCIRT knockdown (KD). (A) Volcano plots showed that 2789 and 2426 genes were significantly upregulated and downregulated in HR condition compared with NC condition, respectively. 1681 and 2476 genes were significantly upregulated and downregulated in KD condition compared with HR condition, respectively. A total of 649 genes were upregulated in HR vs. NC, while being downregulated in KD vs. HR. On the other hand, 381 genes were downregulated in HR vs. NC, while being upregulated in KD vs. HR. (B) Gene ontology enrichment analysis was conducted for above 649 genes in A. (C) Effect of SCIRT knockdown using siRNA2 on VEGFA protein. (D) quantitative analysis of migration assay in Figure 2(b). ^∗^*p* < 0.05,  ^∗∗^*p* < 0.01,  ^∗∗∗^*p* < 0.001, and^∗∗∗∗^*p* < 0.0001. FigS2: the overexpression and knockdown efficiency were evaluated with qRT-PCR. qRT-PCR showed SCIRT was overexpressed by pcDNA3.1-SCIRT (A) and VEGFA was knocked down by siRNA (B). The results are shown as the means ± SD (*n* = 3). *p* < 0.01, compared with control. FigS3: representative images of the tube formation, wound healing, and transwell migration assay. These assays showed that HuR overexpression rescued the SCIRT knockdown-induced inhibitory effect on angiogenesis (A), and HuR knockdown repressed the angiogenesis induced by SCIRT overexpression (B).

Supplementary 2 Table I: siRNA sequence for SCIRT, VEGFA, and HuR. Table II: qRT-PCR primers for SCIRT, VEGFA, and HuR. Table III: HuR targets identified in HUVECs. Table IV: significantly upregulated HuR targets by lncRNA SCIRT knockdown. Table V: significantly downregulated HuR targets by lncRNA SCIRT knockdown. Table VI: normalized mRNA expression values from the microarray data. Table VII: normalized lncRNA expression values from the microarray data. Table VIII: normalized gene expression values from the RNA-seq data.
