# Supplementary material for: Long Noncoding RNA SCIRT Promotes HUVEC Angiogenesis via Stabilizing VEGFA mRNA Induced by Hypoxia
Source: Oxid Med Cell Longev. 2022 Jun 3;2022:9102978. doi: 10.1155/2022/9102978 (PMC9187973; doi:10.1155/2022/9102978)
Supplement: Supplementary 1 — Materials FigS1: transcriptional changes induced by SCIRT knockdown (KD). (A) Volcano plots showed that 2789 and 2426 genes were significantly upregulated and downregulated in HR condition compared with NC condition, respectively. 1681 and 2476 genes were significantly upregulated and downregulated in KD condition compared with HR condition, respectively. A total of 649 genes were upregulated in HR vs. NC, while being downregulated in KD vs. HR. On the other hand, 381 genes were downregulated in HR vs. NC, while being upregulated in KD vs. HR. (B) Gene ontology enrichment analysis was conducted for above 649 genes in A. (C) Effect of SCIRT knockdown using siRNA2 on VEGFA protein. (D) quantitative analysis of migration assay in Figure 2(b). ∗p < 0.05, ∗∗p < 0.01, ∗∗∗p < 0.001, and∗∗∗∗p < 0.0001. FigS2: the overexpression and knockdown efficiency were evaluated with qRT-PCR. qRT-PCR showed SCIRT was overexpressed by pcDNA3.1-SCIRT (A) and VEGFA was knocked down by siRNA (B). The results are shown as the means ± SD (n = 3). p < 0.01, compared with control. FigS3: representative images of the tube formation, wound healing, and transwell migration assay. These assays showed that HuR overexpression rescued the SCIRT knockdown-induced inhibitory effect on angiogenesis (A), and HuR knockdown repressed the angiogenesis induced by SCIRT overexpression (B). [file 9102978.f1.docx]

**Supplementary figures**

**
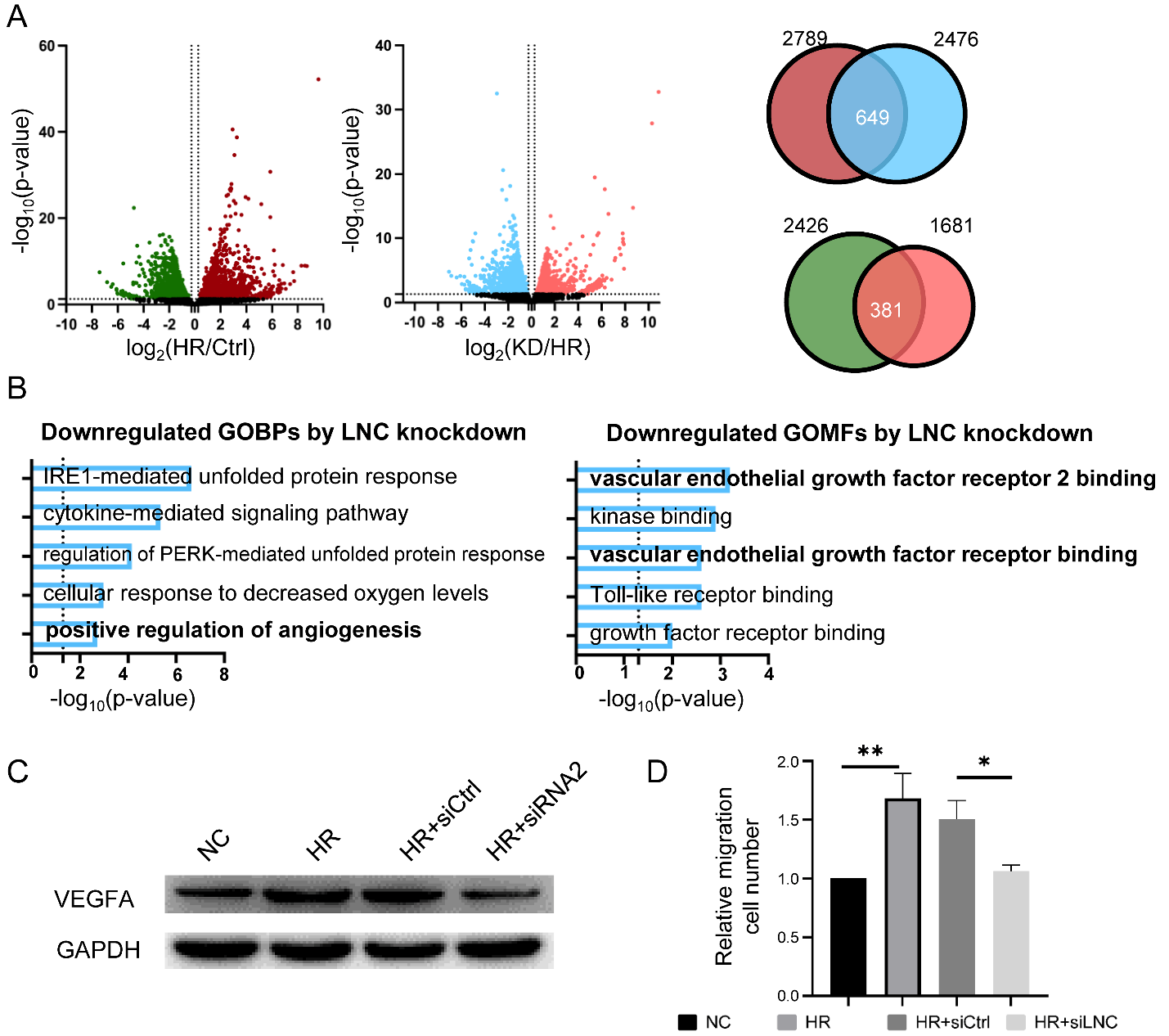
**

**FigS1: Transcriptional changes induced by SCIRT knockdown (KD)**. (A) Volcano plots showed that 2789 and 2426 genes were significantly upregulated and downregulated in HR condition compared with NC condition, respectively. 1681 and 2476 genes were significantly upregulated and downregulated in KD condition compared with HR condition, respectively. A total of 649 genes were upregulated in HR vs. NC, while downregulated in KD vs. HR. on the other hand, 381 genes were downregulated in HR vs. NC, while upregulated in KD vs. HR. (B) Gene ontology enrichment analysis was conducted for above 649 genes in A. (C) Effect of SCIRT knockdown using siRNA2 on VEGFA protein. (D) quantitative analysis of migration assay in Fig 2B. *p < 0.05, **p < 0.01, ***p < 0.001, ****p < 0.0001.

**
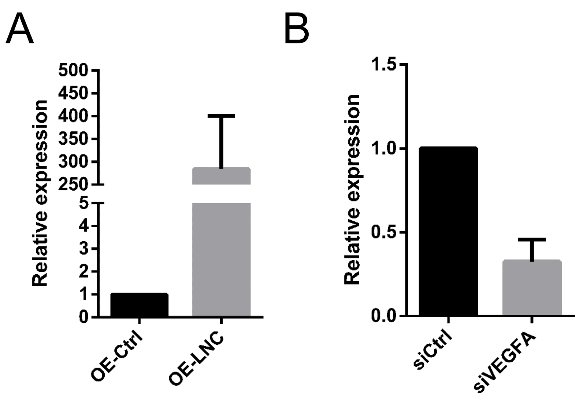
**

**FigS2: The overexpression and knockdown efficiency were evaluated with qPCR.** (A) qPCR showed SCIRT was overexpressed by pcDNA3.1-SCIRT. (B) VEGFA was knocked down by siRNA (B). The results are shown as means ± SD (n = 3). P < 0.01, compared with control.


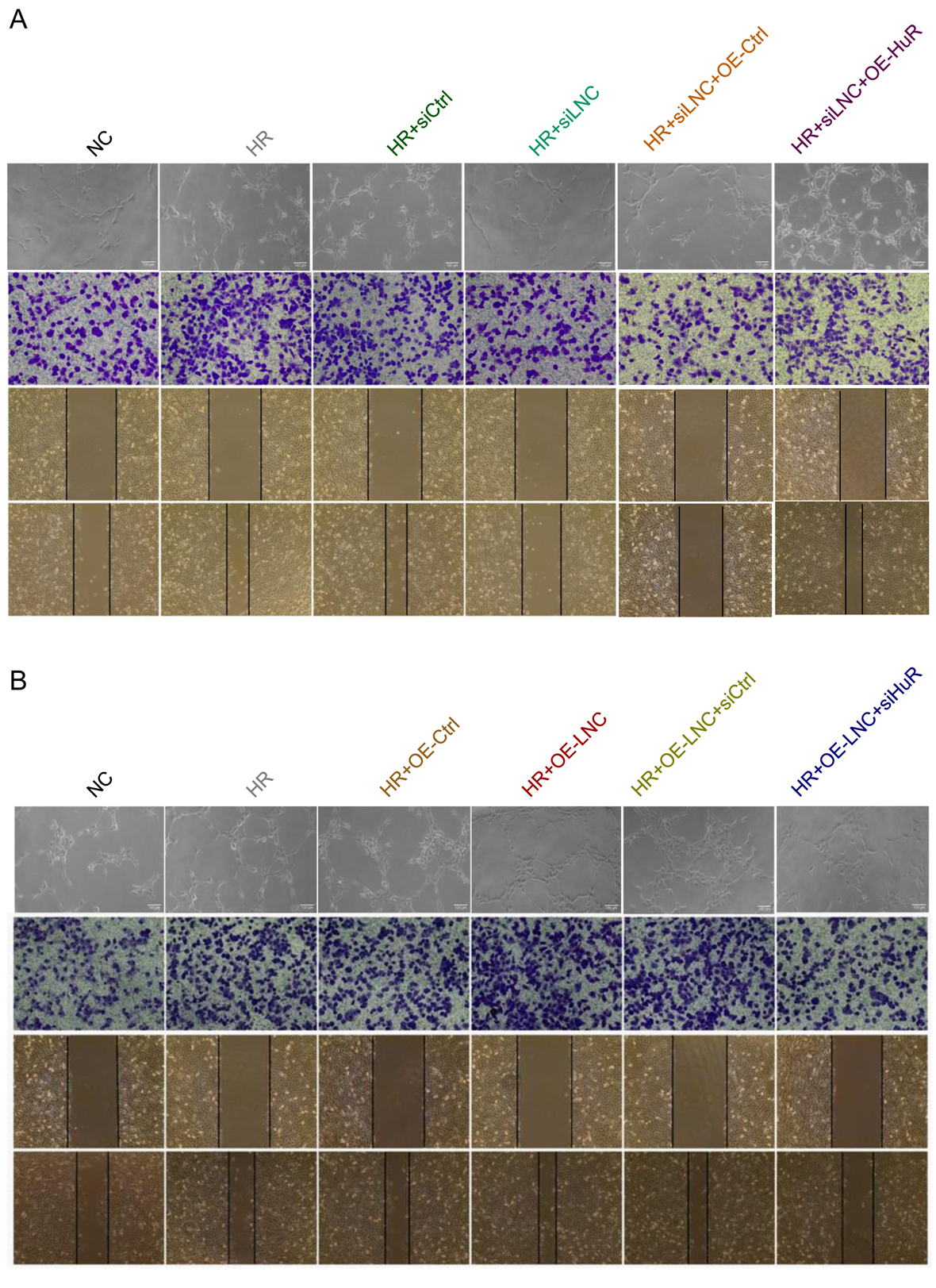


**FigS3: Representative images of the tube formation, wound-healing and transwell migration assay.**

(A) These assays showed that HuR overexpression rescued the SCIRT knockdown-induced inhibitory effect on angiogenesis. (B) HuR knockdown repressed the angiogenesis induced by SCIRT overexpression. (Scale bar, 100 μm)

**Supplementary tables**

Table I. siRNAs sequence for SCIRT, VEGFA and HuR

Table II. qRT-PCR Primers for SCIRT, VEGFA and HuR

Table III. HuR targets identified in HUVECs

Table IV. Significantly upregulated HuR targets by lncRNA SCIRT knockdown

Table V. Significantly downregulated HuR targets by lncRNA SCIRT knockdown

Table VI. Normalized mRNA expression values from the microarray data

Table VII. Normalized lncRNA expression values from the microarray data

Table VIII. Normalized gene expression from values the RNA-seq data
